# Morality in the flesh: on the link between bodily self-consciousness, moral identity and (dis)honest behaviour

**DOI:** 10.1098/rsos.220061

**Published:** 2022-08-31

**Authors:** Marina Scattolin, Maria Serena Panasiti, Salvatore Maria Aglioti

**Affiliations:** ^1^ Sapienza University of Rome and CLN^2^S@Sapienza, Italian Institute of Technology, Rome, Italy; ^2^ Santa Lucia Foundation, IRCCS, Rome, Italy; ^3^ Department of Psychology, Sapienza University of Rome, Rome, Italy

**Keywords:** body self-consciousness, body ownership, sense of agency, moral identity, moral behaviour, honesty

## Abstract

The sense of owning a body (ownership) and controlling its actions (agency) are two main pillars of bodily self-consciousness (BSC). Although studies suggest that BSC signals and morality may be associated, whether such association has a positive or negative direction remains unclear. To investigate this issue, we conducted two pre-registered, online studies, in which a total of 1309 participants completed BSC- and morality-related questionnaires and undertook a task where they could cheat for monetary gain. We found that participants with high sense of ownership displayed high moral identity, which supports the notion that ownership is used to associate the self with positive characteristics. Moreover, high agency was associated with increased moral identity when sense of power is high. Results regarding deception are less clear, and might relate to the impact of COVID-19. Our results concerning moral identity may inspire policies that rely on changes of corporeal awareness to contrast immorality.

## 1. Introduction

Corporeal awareness, or bodily self-consciousness (BSC) [1], is based on two main pillars, namely the feeling of having and owning a body (sense of ownership) [2], and that of being able to initiate and control the said body's movements (sense of agency) [3]. The notion that bodily signals may impact higher-order psychological functions is at the core of embodied cognition theories [4]. What remains largely unknown is whether, or how, different aspects of BSC can influence morality and moral decision making. The relevance of this question for society at large is suggested by the long-documented focus that many religious systems have put into whether, and if so how, awareness of body signals may bias our moral decisions towards dishonesty (e.g. by increasing the temptation from personal rewards) or honesty (e.g. by heightening our sense of responsibility). Despite this, systematic research on whether body ownership and agency—two inherently linked yet distinct constructs [5]—exert different influences on morality is scarce. It is possible, for example, that an increased sense of ownership over the body is associated with higher levels of dishonesty. Indeed, when signals from the body become more prominent, people appear to indulge in different types of self-serving behaviours, such as making more egoistic offers to others during ultimatum games [6,7] or cheating in exchange for rewards [8]. This effect might be driven by the interoceptive component of body ownership which conveys the reward value assigned to certain stimuli and thus may influence consequent decisions [8,9]. In line with this hypothesis, an increase in reward sensitivity (as signalled by enhanced activity in the nucleus accumbens during reward anticipation) seems to be associated with more frequent dishonest behaviours [10]. Conversely, sense of agency may be correlated positively with morality and honest behaviours. For instance, existing literature on intentional binding shows that implicit agency is increased when performing moral actions [11] and reduced when performing immoral ones (i.e. inflicting pain upon others) [12]. Interestingly, the effect of agency on morality might be moderated by such variables as sense of power and moral disengagement. Studies indicate that the perceived sense of power (i.e. the capacity to influence others) [13] may be associated with increased sense of agency, as inferred from intentional binding [14], and to reduced morality as indexed by increased egoistic and unethical behaviours [15]. Moral disengagement refers to different mechanisms through which personal responsibility over immoral deeds is withdrawn and is often associated with higher deception, even at the risk of one's own reputation [16]. Tellingly, moral disengagement is associated with a reduced sense of agency [17]. As such, heightening one's sense of agency may reduce moral disengagement, and thus increase morality.

Importantly, both bodily representations and morality are quintessentially important for the notion of self. Indeed, people perceive that changes in morality-related characteristics alter one's true self more significantly than changes to personality traits, or even memory loss [18,19]. Moral psychology theories posit that self and morality are two systems that become gradually integrated during adolescence [20], and that the internalization of social norms is developmentally important for promoting prosocial behaviour [21]. These integration and internalization processes result in the development of a moral self (i.e. the tendency and ability to care about one's own and others’ ethical conduct) [22]. Previous investigations on whether the moral self can predict actual behaviours has led to contrasting results. Mounting evidence supports the belief that people act in accordance with their moral identity in an effort to confirm their perceived sense of self [23], and that behaviours congruent with this self-concept are more likely to occur when moral values are actively considered (e.g. by being asked to list the Ten Commandments) [24,25] or when such values are perceived as central features of the self [26]. However, other studies have shown that relying on people's self-assessment as moral agents may lead to inaccurate predictions of their behaviour [27]. Indeed, once a moral or immoral concept of self is made salient, people may employ subsequent behaviours to reduce inconsistencies with, and therefore preserve, their moral image [28]. For instance, moral behaviours are more likely to follow negative actions as an attempt to compensate, and thus avoid having to reconsider one's identity—an effect known as moral cleansing [29]. Conversely, once people have proved that they are not immoral or that they are purified, they feel allowed, or licensed, to act less morally [26] and to judge moral violations less severely [30]. Considering all this, we argue that assessing different facets of morality through separate measures may provide a more clear and complete picture of the way in which these facets relate to one another and to other variables.

To investigate the possible link(s) between components of BSC and morality, we conducted a first online study in which we collected self-report measures concerning all variables of interest. This study was completed before the COVID-19 pandemic. Notably, the participants' morality was not only measured using a questionnaire (moral identity) but also by means of a behavioural task where, in one of two blocks, lying was associated with a higher monetary reward. We specifically designed our spot the difference task (STDT) for the study of (dis)honest behaviours in online contexts, and we employed it for the main purpose of investigating whether the senses of ownership and agency are related to (im)moral behaviours and to the moral identity of individuals in similar or different ways. In particular, our initial hypotheses can be summarized as follows:

*H1*: the sense of ownership is negatively associated with moral identity and with moral behaviour;
*H1a*: high reward sensitivity strengthens the negative relationship between sense of ownership and moral identity and between sense of ownership and moral behaviour;
*H2*: the sense of agency is positively associated with moral identity and with moral behaviour;
*H2a*: when sense of power is high, the relationship between sense of agency and moral identity and that between sense of agency and moral behaviour becomes negative; and
*H2b*: low moral disengagement boosts the positive relationship between sense of agency and moral identity and between sense of agency and moral behaviour.

During the COVID-19 pandemic, we then carried out a second study, with the aim of assessing whether the pattern of relationships between BSC components and different measures of morality could be confirmed (or disconfirmed) in an independent sample of participants. We employed the same methods and procedures for both studies, which were approved by the Ethic Committee of the IRCCS Fondazione Santa Lucia (Rome), and were in accordance with the Declaration of Helsinki.

## 2. Study 1

### 2.1. Material and methods

We pre-registered our methods and analysis plan on the Open Science Framework (see https://osf.io/scbnw).

We used RStudio (v. 1.4.1717) [31] to analyse the data: correlations and normality of residuals were computed using functions cor.test and shapiro.test of the *stats* package (v. 4.1.1) [32], respectively. To perform the multiple linear regression and generalized linear mixed effect models regression analysis, we used package *lme4* (v. 1.1-27.1) [33], which was also employed to determine effect sizes. Robust linear regressions were computed using package *MASS*, (v. 7.3-54) [34], while robust linear mixed effect models were performed using package *robustlmm* (v. 2.4-4) [35]. All analyses were two-tailed and all continuous predictors were mean-centred.

#### 2.1.1. Participants

We recruited our participants via Prolific (http://www.prolific.co), and they took part in the study from December 2018 to February 2019—before the widespread effects of the COVID-19 pandemic could bias their responses. In line with general data replicability recommendations [36], our sample (*n* = 658) is consistent with two *a priori* sample size estimations. We performed two power analyses for general linear models via GPower software (v. 3.1.9.2) with *α* = 0.05, a small effect size (0.02) and power of 0.8. The results indicate that 550 and 647 responses were needed for three and five predictors, respectively.

A total of 705 participants took part in the study, 47 of whom were excluded from the final dataset (the full data for both reasons and occurrences are reported in the electronic supplementary material, table S1). Our final sample included 658 participants (females = 314) between the ages of 18–66 (*M* = 29.37, s.d. = 9.68), who declared compliance with the requirements of the study (i.e. not having any neurological or psychiatric condition, nor taking any psychiatric drugs) and who passed at least one of the two attention-check questions. Italian was the first language of all the participants. All the participants gave their informed consent and were paid for their participation.

#### 2.1.2. Materials

We asked the participants to complete a series of questionnaires measuring different components of BSC, with the intent of assessing their possible link with moral identity and behaviour, as well as exploring which other variables could modulate this relation. All questionnaires, and the online behavioural task, were developed using PsyToolkit (v. 2.5.4) [37]. Additional information on the measures used in the study can be found in the electronic supplementary material.

#### 2.1.3. Morality measures

##### 2.1.3.1. Moral identity

We assessed the moral identity of participants using the moral identity measure [38]. This questionnaire is composed of items loading on two factors, namely symbolization (e.g. ‘I often buy products that communicate the fact that I have these characteristics'; *α* = 0.73) and internalization (e.g. ‘Being someone who has these characteristics is an important part of who I am'; *α* = 0.79). Owing to our aim being that of investigating individuals' moral self-concept (as measured by factor internalization), and not the way in which these notions of self are conveyed to others (factor symbolization), our measure of moral identity relied exclusively on the internalized subscale of the questionnaire (see the electronic supplementary material, table S2 for the list of items included in this subscale).

##### 2.1.3.2. (Im)moral behaviour

To assess the (im)moral behaviour of our participants, we used a version of the STDT in which participants were presented with pairs of images in the middle of the screen, and asked to find as many differences as possible within 45 s. A countdown was displayed on the top centre of the screen while numbers from 0 to 10 were shown on the bottom. Once the countdown had ended, the participants were instructed to click on the number corresponding to the differences they had found. The participants were informed that 10 differences existed between the image pairs and that, given the same amount of time, most people found only five of these. All participants completed the task twice under two different conditions (no reward and reward), the order of which was randomized.

Each condition was completed with one of two possible image pairs. We used a web search to obtain these, and other, images. We based the selection of the two image pairs employed in the STDT on a validation study (see the electronic supplementary material section for a full description). For every participant, each image pair was randomly assigned to only one experimental condition.

In the no reward condition, participants gained €0.10 for completing the task; in the reward condition, further to the aforementioned €0.10, participants received an additional €0.30 for each difference above five they reported finding, plus a further €10 if, at the end of data collection, they were among those who found the highest number of differences. Crucially, the images in each pair only differed in five details, so any response above this number was considered a lie. Thus, participants who, in the reward condition, behaved dishonestly and reported more than the actual number of existing differences, received a higher monetary pay-off when compared with honest participants. This choice was aimed at inducing a real conflict between following social norms and prioritizing personal (in this case, monetary) profits. Such conflict is believed to be a core aspect of the process leading to (dis)honest decisions [39] and its induction is common practice in the field of morality research [16,40–45]. Lies were calculated by subtracting the actual number of differences from the responses (results smaller than, or equal to, zero were coded as zero). Participants also had the opportunity to enter their responses after the countdown had ended. Indeed, both the images and the response options continued to be displayed after the countdown.

**Figure 1.**
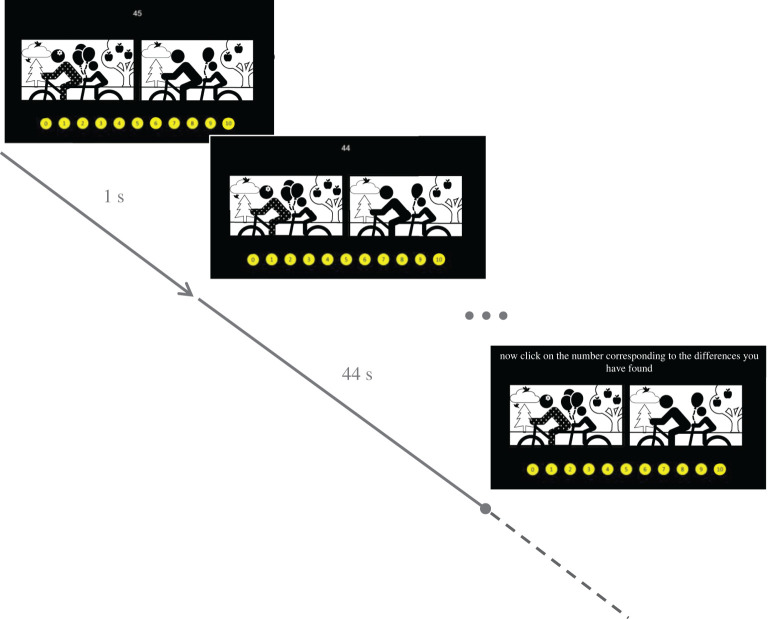
Example of spot the difference task (STDT) trial. The task was composed of two trials, which were completed by all participants under two different conditions—no reward and reward. In both of the STDT trials, participants were asked to compare a pair of images in order to find as many differences as they could in 45 s. Participants were told that the images contained 10 details, yet the majority of people could only find five in the given timeframe. In the reward condition (versus no reward condition) participants obtained €0.30 for each difference above five they reported finding. At the end of the data collection process, the participants who identified the highest number of differences received an additional €10. Crucially, and unbeknownst to the participants, only five differences existed between the images. Any response above five could then be considered a lie. A countdown signalling the remaining time (in seconds) was displayed on the top-centre of the screen. At any time, participants could indicate the number of identified differences by clicking on the corresponding number within the yellow dots. Once the countdown hit zero, the participants could still compare the two images, as these remained on-screen until a response was provided. The dashed line represents a possible delayed response. Owing to copyright regulations, the above figure shows a mock pair of images not shown during the study. The images presented here were created by the authors for the sole purpose of exemplifying the STDT.

By way of example, and owing to copyright regulations, figure 1 shows a mock pair of images specifically created by the authors for the purpose of publication.

It is worth noting that different versions of the STDT were recently used to investigate dishonesty [46,47] and unveil its underlying neural correlates [48]. Crucially, this task has the advantage of being nonverbal, which allows us to investigate moral behaviours in populations differing in age and education. This is particularly relevant to the two studies presented here, where we planned to recruit two large samples of participants that therefore varied in age and schooling. Additionally, the instructions of the STDT may not be totally novel to participants, as they resemble those found in popular puzzle games and could therefore be easier to grasp. However, our paradigm differs from the versions employed in prior studies in that it required the participants to report the number of differences they (allegedly) identified, and not only whether they had or had not found all of the (supposedly) present differences. This provided us with the opportunity of measuring dishonesty along a continuum, where reporting a higher number constituted a more serious moral violation as it deviated more from the reality presented to our participants.

#### 2.1.4. Body self-consciousness measures

We used two scales to measure sense of ownership, namely the ownership subscale of the Embodied Sense of Self Scale (ESSS) [49] (e.g. ‘Sometimes the clothes I am wearing feel heavy', *α* = 0.73) and the private body subscale of the Body Consciousness Questionnaire (BCQ) [50] (e.g. ‘I am sensitive to internal bodily tensions'; *α* = 0.65). Specifically, the BCQ has a three-factor structure, namely private body consciousness (e.g. ‘I am sensitive to internal bodily tensions'), public body consciousness (e.g. ‘When with others, I want my hands to be clean and look nice'), and body competence (e.g. ‘I'm capable of moving quickly'). Owing to our specific research interest in the interoceptive component of ownership, we only included the five items of the private body subscale (*α* = 0.65) in the survey. Additional information concerning the ESSS is reported in the electronic supplementary material, and all items included in the private body subscale of the BCQ are presented in the electronic supplementary material, table S3.

As stated in the pre-registration, participants' responses to the two ownership questionnaires were to be combined into a single measure after reversing responses to the ESSS. In fact, while the BCQ measures interoceptive awareness, higher ESSS scores reflect a more anomalous sense of ownership, such as that observed in schizophrenia [49]. Although we expected these two measures to be negatively correlated, their Pearson's correlation coefficient was positive (*r* = 0.16, 95% confidence interval (CI) [0.09, 0.24], *p* < 0.001; table 1), which indicated that the two could not be combined. Previous studies have suggested that interoception modulates the degree to which a virtual hand or body is perceived as belonging to the self [51–54], thereby supporting the notion that—although not sufficient—inner body signals are necessary for sense of ownership [55]. Consequently, we only used BCQ scores in those models with body ownership as a predictor. This constitutes a deviation from the analysis plan described in our pre-registration; therefore, all models testing the role of body ownership by means of BCQ should be considered exploratory.

**Table 1.** Pearson's correlation coefficients (*r*) between demographic variables, ownership questionnaires and morality measures collected during study 1. (Age, education and subjective economic status (SES) are the demographic variables. The sense of ownership questionnaires are the body consciousness questionnaire or BCQ, and the embodied sense of self scale or ESSS. The moral identity scores and lies in the spot the difference task are the two measures of morality. Values in square brackets represent 95% confidence intervals. Asterisks indicate significance: *p < 0.05, ***p < 0.001.)

variable	1	2	3	4	5	6
1. age						
2. education	0.25***					
	[0.17, 0.32]					
3. SES	−0.17***	0.13***				
	[−0.24, −0.09]	[0.06, 0.21]				
4. BCQ	0.07	−0.02	−0.09*			
	[−0.01, 0.14]	[−0.10, 0.05]	[−0.17, −0.01]			
5. ESSS	−0.04	−0.06	−0.09*	0.16***		
	[−0.11, 0.04]	[−0.14, 0.01]	[−0.16, −0.01]	[0.09, 0.24]		
6. moral identity	0.03	0.09*	0.00	0.15***	−0.08*	
	[−0.05, 0.11]	[0.01, 0.16]	[−0.07, 0.08]	[0.08, 0.23]	[−0.16, −0.01]	
7. lies	0.08*	−0.03	−0.01	0.03	0.09*	−0.10*
	[0.001, 0.15]	[−0.11, 0.04]	[−0.09, 0.07]	[−0.05, 0.10]	[0.01, 0.17]	[−0.17, −0.02]

For sense of agency, we employed the positive agency subscale from Tapal and colleagues (e.g. ‘I am in full control of what I do') [56]. This showed positive internal consistency (*α* = 0.78).

#### 2.1.5. Moderating variables

Considering that the task included a financial reward, we used the Monetary Intelligence Scale [57] as a measure of reward sensitivity, which consists of the affective (*α* > 0.85), behavioural (*α* > 0.7) and cognitive (*α* > 0.85) sub-constructs. The eight-item Sense of Power Scale [13] (*α* = 0.85) and Propensity to Morally Disengage Scale [58] (*α* = 0.77) were used to assess sense of power and moral disengagement, respectively.

#### 2.1.6. Procedure

The participants could complete all parts of the survey using any computer with an internet connection. We also provided them with general information regarding the study. In the consent page, participants had to tick a box to indicate their agreement to participation—not doing so would bar them from completing the subsequent steps. Demographic information (i.e. age, sex, highest education degree received, subjective economic status (SES)) was collected prior to the STDT. To assess SES, we asked the participants to place themselves along a Visual Analogue Scale, ranging from ‘People who have the least money’ to ‘People who have the most money’ (scores ranged from 0 to 100). This method was adapted from Adler *et al*. [59] and Ostrove *et al*. [60].

Questionnaires followed the task. The order of conditions and pairs of images in the STDT was randomized, as was the order of questionnaires and the questions themselves. The Monetary Intelligence Scale and Sense of Agency Scale included one attention-check question each, namely ‘answer ‘agree’ to this sentence’ and ‘the answer to this question should be ‘disagree’’. The entire study could be completed in approximately 20 min.

### 2.2. Results of exploratory analysis

In the present section, we report all analyses concerning the link between each of our morality measures and body ownership, as assessed exclusively by means of the BCQ.

To control for the role of potential confounders, we ran additional analysis and included specific demographic variables as fixed covariates to all of the regression models reported in this section. Such demographic variables were selected based on their significant correlation with the dependent variable under consideration. Because the body ownership measure could not be computed according to our pre-registration and because our original analysis plan did not include covariate(s) (see https://osf.io/scbnw), all regression models included in this section should be considered exploratory. For the results of planned regression models investigating the role of sense of agency, see the electronic supplementary material.

#### 2.2.1. Moral identity

We analysed moral identity by using two multiple linear regression models. First, we checked whether any demographic variable (i.e. age, sex, education, or SES) was associated with moral identity. Owing to education being significantly correlated (*r* = 0.09, 95% CI [0.01, 0.16], *p* = 0.023; table 1), we included it in both models as a covariate.

##### 2.2.1.1. The relationship between sense of ownership and moral identity

**Figure 2.**
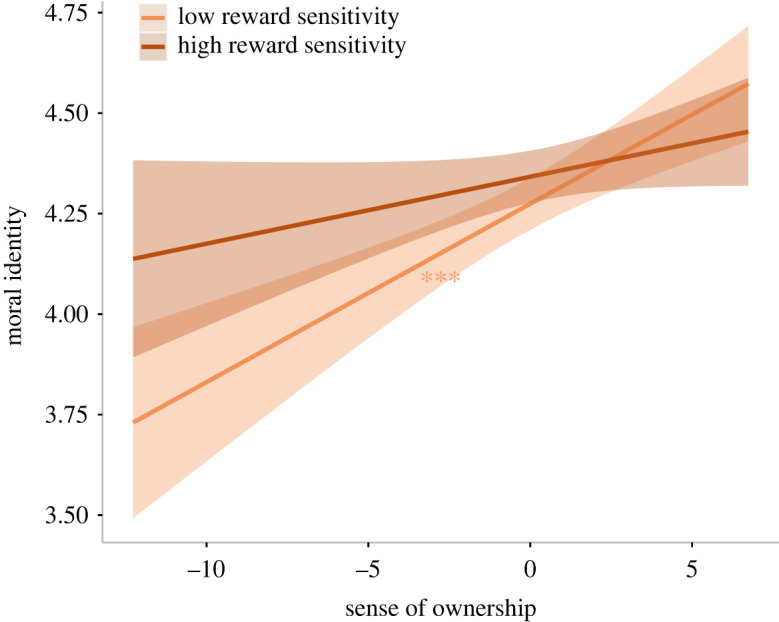
Moral identity scores as a function of ownership and reward sensitivity (study 1). The plot shows regression lines for 1 s.d. below (low reward sensitivity) and above (high reward sensitivity) the mean reward sensitivity score of study 1. The shaded bands represent 95% confidence intervals, and the asterisks indicate significance. ***p < 0.001.

To investigate whether moral identity was predicted by body ownership, as well as if this effect was moderated by sensitivity to rewards, we entered these two variables and their interaction term as fixed factors in a model predicting moral identity. As residuals were significantly non-normal (*W* = 0.94, *p* < 0.001), we computed (and here present) a robust regression. Sense of ownership was associated with a significant increase of moral identity (*β* = 0.03, 95% CI = [0.02, 0.04], *t*_653_ = 4.46, *p* < 0.001), and the interaction between ownership and reward sensitivity was significant (*β* = −0.06, 95% CI = [−0.11, −0.01], *t*_653_ = −2.28, *p* = 0.023; figure 2). While sense of ownership is positively associated with moral identity when reward sensitivity is low (*β* = 0.05, 95% CI = [0.03, 0.07], *t*_653_ = 4.28, *p* < 0.001; figure 2), the slope for high reward sensitivity is not significantly different from 0 (*β* = 0.02, 95% CI = [−0.01, 0.04], *t*_653_ = 1.18, *p* = 0.238; figure 2). Further information regarding the results of this regression model are reported in the electronic supplementary material, table S4.

##### 2.2.1.2. The relationship between sense of agency and moral identity

**Figure 3.**
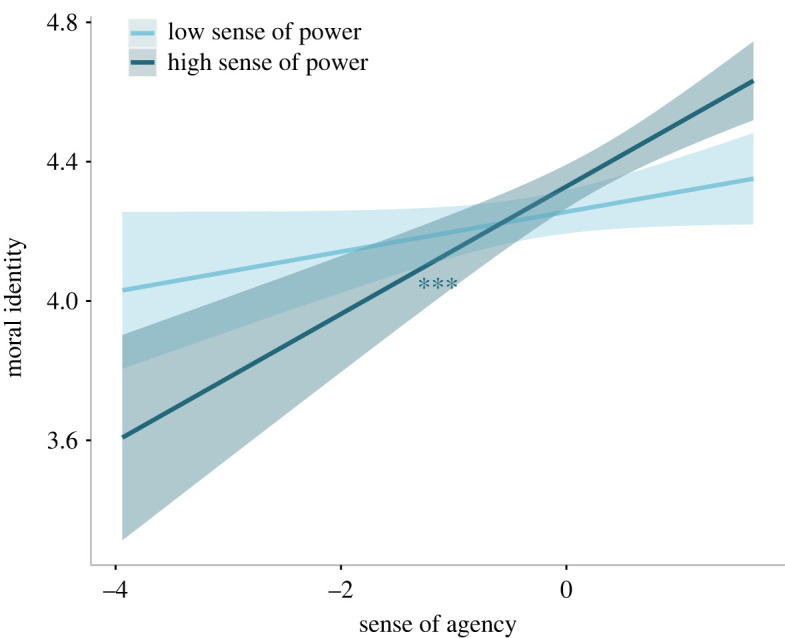
Moral identity scores as a function of agency and sense of power (study 1). The plot shows regression lines for 1 s.d. below (low sense of power) and above (high sense of power) the mean sense of power score of study 1. The shaded bands represent 95% confidence intervals and the asterisks indicate significance. ***p < 0.001.

**Figure 4.**
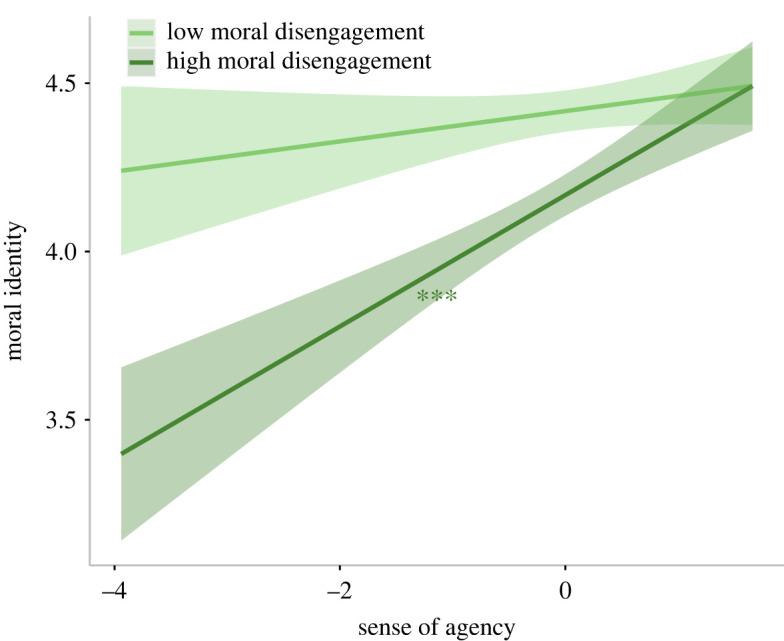
Moral identity scores as a function of agency and moral disengagement (study 1). The plot shows regression lines for 1 s.d. below (low moral disengagement) and above (high moral disengagement) the mean moral disengagement score of study 1. The shaded bands represent 95% confidence intervals and the asterisks indicate significance. ***p < 0.001.

In the second model, moral identity was the dependent variable while sense of agency, sense of power and moral disengagement were the independent variables. To analyse whether sense of power and/or moral disengagement could moderate the effect of agency, we entered two interaction terms as fixed factors (sense of agency × sense of power and sense of agency × moral disengagement). We performed a robust regression after a Shapiro–Wilk test showed a significant deviation of residuals from normality (*W* = 0.95, *p* < 0.001). The robust results show that sense of agency was significantly and positively associated with moral identity (*β* = 0.12, 95% CI = [0.07, 0.17], *t*_651_ = 4.98, *p* < 0.001), while moral disengagement was negatively associated with it (*β* = −0.15, 95% CI = [−0.21, −0.10], *t*_651_ = −5.64, *p* < 0.001). The interaction between agency and sense of power was significant (*β* = 0.07, 95% CI = [0.02, 0.11], *t*_651_ = 2.77, *p* = 0.006; figure 3), in that an increase of agency predicts higher moral identity when sense of power is high (*β* = 0.17, 95% CI = [0.09, 0.25], *t*_651_ = 4.01, *p* < 0.001; figure 3). Also significant was the interaction between agency and moral disengagement (*β* = 0.09, 95% CI = [0.04, 0.15], *t*_651_ = 3.41, *p* < 0.001), which indicated that agency positively predicts moral identity only when moral disengagement is high (*β* = 0.17, 95% CI = [0.09, 0.26], *t*_651_ = 3.87, *p* < 0.001; figure 4). The complete results are reported in the electronic supplementary material, table S5.

#### 2.2.2. (Im)moral behaviour

To ensure that the two pairs of images did not differ in terms of how many details the participants reported finding, we first ran a multiple linear mixed effect model. Lies were the predicted variable, while participants' identification number (ID) and the presented pair of images were entered as the random and fixed factor, respectively. This analysis revealed that the specific pair of images did not predict the number of differences reported by participants (*β* = 0.03, 95% CI = [−0.03, 0.10], t_655.32_ = 0.99, *p* = 0.323). A detailed description of participants’ responses in the no reward and reward conditions across the two pairs of images is provided in table 2.

**Table 2.** Distribution of responses for each experimental condition of the spot the difference task (STDT) (study 1). (‘%’ indicates the percentage of participants who reported the corresponding number of differences. Responses between 0 and 5 count as the same response, as they could not be considered as lies and were thus coded as zero.)

no reward condition		reward condition	
differences reported	(%)	differences reported	(%)
between 0 and 5	87.50	between 0 and 5	82.85
6	10.98	6	11.02
7	0.61	7	2.76
8	0.91	8	1.99
9	0	9	0
10	0	10	1.38

To investigate whether sense of ownership and agency can predict dishonest behaviour, as well as whether other variables acted as moderators, we used three additional multiple linear mixed effect models. In all of these analyses, participants' IDs were set as random factors, while their ages were included as fixed predictors (as it significantly correlated with the variable lies; *r* = 0.08, 95% CI [0.001, 0.15], *p* = 0.049; table 1).

##### 2.2.2.1. The relationship between sense of ownership and (im)moral behaviour

**Figure 5.**
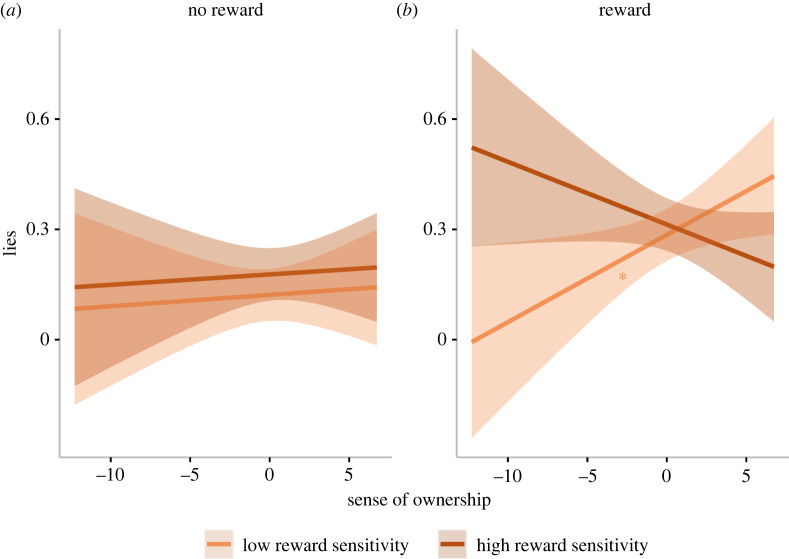
Lies as a function of ownership, reward sensitivity and experimental condition (study 1). (*a*) Lies in the no reward condition, while the reward condition is displayed in (*b*). The plot shows regression lines for 1 s.d. below (low reward sensitivity) and above (high reward sensitivity) the mean reward sensitivity score. The shaded bands represent 95% confidence intervals. Asterisks indicate significance *p < 0.05.

In the first of these models, we entered body ownership, experimental condition (no reward coded as 0, reward coded as 1), reward sensitivity, and all of the interaction terms, as fixed predictors. A Shapiro–Wilk test showed that residuals did not follow a normal distribution (*W* = 0.55, *p* < 0.001), thus we entered the same exploratory model in a robust mixed regression analysis. Given the similarity of robust mixed regression results and multiple linear mixed effect model, only the latter—which allows *post hoc* comparison testing for mixed effects models—will be reported here. Robust results can be found in the electronic supplementary material. We observed that the experimental condition (no reward/reward) came with an increase in the number of lies (*β* = 0.15, 95% CI = [0.08, 0.21], *t*_652.40_ = 4.50, *p* < 0.001). The interaction between ownership, condition and reward sensitivity is significant (*β* = −0.09, 95% CI = [−0.17, −0.01], *t*_651.21_ = −2.24, *p* = 0.025; figure 5). Indeed, in the reward condition, sense of ownership was positively associated with the number of lies in participants who scored low in reward sensitivity (*β* = 0.02, 95% CI = [0.003, 0.04], *t*_1271.73_ = 2.26, *p* = 0.024; figure 5). Further information regarding the results of this regression model are reported in the electronic supplementary material, table S6.

##### 2.2.2.2. The relationship between sense of agency and (im)moral behaviour

We computed two models so as to examine the possible link between lies and sense of agency. Agency, experimental condition (no reward; reward) and sense of power were set as independent variables in one of these models. To investigate possible moderations, we included their interaction (agency × experimental condition × sense of power) in this analysis. A Shapiro–Wilk test showed that the distribution of residuals was significantly non-normal (*W* = 0.54, *p* < 0.001), leading us to perform a robust regression analysis on the same exploratory model. The robust analysis found that the number of reported differences increased in association with age (*β* = 0.001, 95% CI = [0.00, 0.01], *t*_650.67_ = 3.43, *p* < 0.001), yet no other predictor was significant. The complete results are reported in the electronic supplementary material, table S7.

In the last model, sense of agency, experimental condition (no reward; reward), moral disengagement and all interactions between these (agency × experimental condition × moral disengagement) were fixed factors. We computed a robust multiple linear mixed effect model owing to the significantly non-normal residuals (*W* = 0.56, *p* < 0.001). The robust results show that the number of lies in the task increased in association with the experimental condition (no reward/reward) (*β* = 0.001, 95% CI = [0.00, 0.01], *t*_652.92_ = 2.46, *p* = 0.014) and age (*β* = 0.001, 95% CI = [0.00, 0.01], *t*_650.63_ = 3.51, *p* < 0.001). All other predictors were not significant. Further information regarding the results of this model is presented in the electronic supplementary material, table S8.

### 2.3. Discussion

The results of exploratory analyses on the data of this first study show that both components of BSC, namely the sense of ownership and the sense of agency, predict a significant increase of moral identity. While results concerning the latter appear to confirm our hypothesis *H2*, the relationship between the sense of ownership and moral identity seems to go in the opposite direction with respect to what we had anticipated with hypothesis *H1*. Additionally, we found that other variables are associated with each specific BSC component and with morality. In particular, we observed that, contrary to what we had expected (see hypothesis *H1a*), as the sense of ownership intensifies, it is *low* reward sensitivity that becomes associated with increased moral identity (figure 2) and, concurrently, with increased deceptive behaviour during the STDT (figure 5). This (seemingly paradoxical) result may be in keeping with previous studies which indicate that, when people perceive themselves as highly moral, they feel more licensed to act less morally—an effect referred to as moral licensing [26,28]. It is then possible that awareness of one's own body may enhance moral self-image, which in turn may steer one towards dishonesty. Interestingly, we observed no such pattern for high levels of reward sensitivity. Indeed, the above effect tended to occur when an enhanced sense of body ownership conveyed signals of low reward sensitivity. Traditionally speaking, the way in which reward sensitivity influences reward-related behaviours (e.g. addiction) has been believed to be nonlinear [61]. According to the Reward Deficiency Model [62], low sensitivity to reward encourages people to seek bigger rewards in order to boost a depressed dopamine system. Concurrently, however, similar reward-seeking behaviours are observed in association with hypersensitivity to rewards [63,64].

Albeit opposite to what we had anticipated (see hypothesis *H2a*), another interesting result is that sense of agency is *positively* (and not negatively) related with moral identity in participants who reported being able to influence others, that is, in conditions of high sense of power. Lammers and Stapel demonstrated that feeling powerful is associated with a preference for deontological moral judgements, which may in turn enforce a moral concept of self [65]. However, we found that the interaction between the senses of agency and power were not significantly associated with participants' behaviour during the task.

Surprisingly, given what we had hypothesized *a priori* (see *H2b*), agency is positively associated with moral identity in people who employ justification strategies for their wrongdoings. This suggests that feeling in control of one's own actions can partly counterbalance the negative effect of moral disengagement. Specifically, self-sanction mechanisms may be crucial to this process, especially when the sense of agency is high. In fact, such mechanisms are activated to prevent behaviours that are inconsistent with each individual's moral standards and that are difficult to justify [66]. While sense of agency and moral disengagement were associated with moral identity, their relationship to behaviour in a given task does not appear to be significant.

## 3. Study 2

Since some exploratory analysis were included in study 1, we decided to conduct a pre-registered replication study to confirm the significance of our effects.

### 3.1. Material and methods

The methods and analysis plan for this replication study were pre-registered on the Open Science Framework (https://osf.io/tnp7g) prior to data collection.

Version 1.4.1717 of RStudio [31] was used for all analysis presented here. We employed the same functions within packages *stats* (v. 4.1.1) [32], *lme4* (v. 1.1-27.1) [33], *MASS* (v. 7.3–54) [34] and *robustlmm* (v. 2.4-4) [35] with the same purposes reported in the corresponding section of study 1. Slope comparisons were performed using function *emmeans* within the homonymous package (v. 1.6.2-1). As for the first study, all analyses were two-tailed and all continuous predictors were mean-centred.

#### 3.1.1. Participants

Recruitment of participants occurred through Prolific (http://www.prolific.co) between July and August 2021, when 703 individuals took part in this study. We excluded the responses of 52 of these participants for the reasons and number of occurrences described in the electronic supplementary material, table S9.

Our final sample consists of 651 individuals (females = 285; preferred not to answer this question = 3) between 18 and 66 years of age (*M* = 27.58, s.d. = 8.12), whose first language was Italian and who did not participate in study 1. The participants passed at least one of the attention-checks and reported they were not taking any psychiatric medication nor had been diagnosed with psychiatric and/or neurological disorder(s) at the time of participation.

The numerosity of the final sample is in line with the results of four independent power analyses computed with GPower software (v. 3.1.9.7) and based on the effect sizes we observed in study 1. We included a detailed description of these analyses in the electronic supplementary material.

#### 3.1.2. Materials

We asked the participants of study 2 to complete the same morality, BSC and moderating measures we employed in study 1. The data collected in study 2 also show a significant, positive correlation between the BCQ and ESSS scores (*r* = 0.23, 95% CI [0.15, 0.30], *p* < 0.001), thus confirming that these two scales could not be combined into a single measure of body ownership. Therefore, and as in study 1, all analyses where body ownership is set as a fixed predictor rely exclusively on BCQ scores.

Additionally, as declared in the pre-registration form, participants answered a series of questions concerning their COVID-related experiences. This was done to assess whether possible differences between the results of our two studies could be attributed to the pandemic situation, which could not have played any role in study 1. Considering this, we asked participants to: (i) report whether they had ever tested positive for *SARS-CoV-2*; (ii) report whether they were presenting COVID-19 symptomatology at the time of participation; (iii) rate how dangerous they believed getting COVID-19 would be for them; and (iv) rate how likely they were to get vaccinated against COVID-19.

All parts of this study were presented to participants using the web version of PsyToolkit (3.3.2) [37].

#### 3.1.3. Procedure

The participants of study 2 followed the same procedure as study 1. To avoid biasing participants' responses to those measures included in the original study, all the additional, COVID-related questions were presented after all other measures had been collected.

### 3.2. Results of pre-registered analyses

To assess whether the data collected during study 2 replicated the results of our first study, we analysed moral identity and (im)moral behaviour with the same multiple linear (mixed) models we described in the exploratory analysis section of study 1.

#### 3.2.1. Moral identity

##### 3.2.1.1. The relationship between sense of ownership and moral identity

We investigated the link between moral identity, body ownership and reward sensitivity by means of a multiple linear regression model. We set moral identity as the dependent variable, while body ownership, reward sensitivity and their interaction were the fixed predictors. As in study 1, we included education as a fixed covariate.

Because residuals were significantly non-normal (*W* = 0.93, *p* < 0.001), we report here the results of a robust regression. We observed that sense of ownership was associated with a significant increase of moral identity (*β* = 0.03, 95% CI = [0.02, 0.05], *t*_646_ = 5.35, *p* < 0.001). No other factor was significant. The complete results of this regression model is reported in the electronic supplementary material, table S10.

##### 3.2.1.2. The relationship between sense of agency and moral identity

Moral identity was the predicted variable in a model assessing its relationships with the sense of agency. We included the latter as fixed factor in a multiple linear model, together with sense of power and moral disengagement. The interaction between sense of agency and sense of power and between sense of agency and moral disengagement were also entered as fixed predictors, while education was set as a covariate. As residuals significantly deviated from normality (*W* = 0.93, *p* < 0.001), we ran (and present here) a robust linear regression.

**Figure 6.**
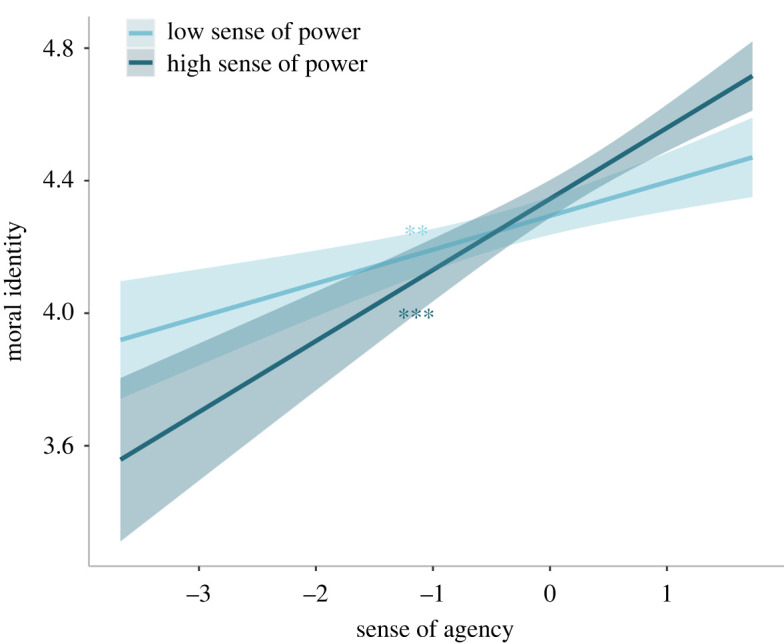
Moral identity scores as a function of agency and sense of power (study 2). The plot shows regression lines for 1 s.d. below (low sense of power) and above (high sense of power) the mean sense of power score observed in study 2. The shaded bands represent 95% confidence intervals and the asterisks indicate significance. **p < 0.01, ***p < 0.001.

While the sense of agency was significantly and positively related with moral identity (*β* = 0.16, 95% CI = [0.11, 0.20], *t*_644_ = 6.65, *p* < 0.001), an increase of moral disengagement is associated with a significant reduction of moral identity scores (*β* = −0.10, 95% CI = [−0.16, −0.05], *t*_644_ = −3.74, *p* < 0.001). The interaction between the senses of agency and power was also significant (*β* = 0.06, 95% CI = [0.02, 0.09], *t*_644_ = 3.00, *p* = 0.003). *Post hoc* analyses revealed that an increase of sense of agency is associated with a significant increase in moral identity when the sense of power is low (*β* = 0.10, 95% CI = [0.03, 0.17], *t*_644_ = 2.86, *p* = 0.004; figure 6) and also when the sense of power is high (*β* = 0.22, 95% CI = [0.15, 0.29], *t*_644_ = 6.19, *p* < 0.001; figure 6). However, when sense of power is high, the positive association between moral identity and sense of agency is significantly higher compared to when sense of power is low (estimate = 0.21, 95% CI = [0.07, 0.35], z.ratio = 3.00, *p* = 0.032). More information regarding the results of this regression model can be found in the electronic supplementary material, table S11.

#### 3.2.2. (Im)moral behaviour

Prior to any other analysis, we checked whether the participants had reported similar numbers of differences between the two pairs of images of the STDT. To this purpose, we ran a multiple linear mixed effect model with lies as the dependent variable. The pair of images presented to the participants was the fixed predictor while the participants’ IDs were the random factor. Our results confirm that the pairs of images employed in this study were similar in terms of the number of differences the participants reported finding (*β* = −0.06, 95% CI = [−0.13, 0.01], *t*_650.00_ = −1.66, *p* = 0.098). In table 3, we report, for each of the possible responses to the task, the proportion of participants who provided that specific response.

**Table 3.** Distribution of responses for each experimental condition of the spot the difference task (STDT) (study 2). (‘%’ indicates the percentage of participants who reported the corresponding number of differences. Responses between 0 and 5 count as the same response, as they could not be considered as lies and were thus coded as zero.)

no reward condition		reward condition	
response	(%)	response	(%)
between 0 and 5	90.00	between 0 and 5	84.02
6	6.91	6	12.29
7	1.38	7	1.08
8	0.61	8	0.77
9	0.15	9	0
10	0.92	10	1.84

##### 3.2.2.1. The relationship between sense of ownership and (im)moral behaviour

To analyse the relationship between the participants' (im)moral behaviour and their sense of ownership, as well as the role of reward sensitivity, we used a multiple linear mixed effect model. Specifically, we set lies as the predicted variable, while body ownership, reward sensitivity, the experimental condition (no reward coded as 0, reward coded as 1) and all their interaction terms were the fixed predictors. The age of the participants was included as a fixed covariate, while the participants’ IDs were entered as a random factor.

The results of this analysis show that the participants reported more lies during the reward condition compared to the no reward condition (*β* = 0.09, 95% CI = [0.02, 0.16], *t*_647.00_ = 2.53, *p* = 0.012). No other effect or interaction was significant (as can be seen in the electronic supplementary material, table S12). Following a significant Shapiro–Wilk test (*W* = 0.49, *p* < 0.001), we ran a robust version of the same model. Because the two analysis show similar results, we report the robust version in the electronic supplementary material.

### 3.3. Results of exploratory analysis

Considering that our second study has been conducted during the COVID-19 pandemic, we ran some exploratory analysis to assess whether any of our COVID-related measures was associated with the sense of ownership of the participants. We observed that sense of body ownership significantly and positively correlated only with how dangerous the participants thought contracting SARS-CoV-2 would be for themselves (*r* = 0.12, 95% CI [0.04, 0.20], *p* = 0.002).

### 3.4. Discussion

The data of our second study appear to confirm the existence of a relationship between moral identity and sense of ownership and agency. Indeed, and as we also observed in study 1, an increase in the sense of ownership came with a stronger moral identity. Similarly, we found that high levels of sense of agency are associated with higher moral identity scores. Moreover, in line with study 1, the relationship between sense of agency and moral identity was stronger for those who were higher on sense of power.

Contrary to what we observed in our first study, we did not find any evidence that reward sensitivity could modulate the association between body ownership and the participants' moral identity or their (im)moral behaviour during the STDT. In fact, we found that only the experimental condition (i.e. no reward/reward) was associated with an increase of the number of lies.

## 4. General discussion

We investigated whether two of the basic pillars of BSC (the senses of ownership and agency) are differentially related to moral identity and (im)moral behaviour. Specifically, we tested if sensitivity to rewards could modulate the effect of body ownership, while concurrently exploring whether moral disengagement and sense of power could impact the effect of the sense of agency. To answer these questions, we conducted an online study and a pre-registered replication study, where moral identity was measured by means of a questionnaire and (im)moral behaviour was measured by a task that tempted participants to lie in exchange for a higher monetary reward.

Interestingly, we found that both sense of ownership and sense of agency were significantly and positively associated with moral identity in two independent samples of participants. The relationship between moral identity and sense of agency is in line with our hypothesis *H2* and may possibly be explained through personal responsibility. In fact, sense of agency over the body entails feelings of responsibility over the outcomes of performed actions [11]. Feeling responsible for the effects of one's own behaviours may thus promote the occurrence of moral deeds [66] and, by consequence, consolidate one's moral concept of the self. In line with this, we found that acting dishonestly was associated with decreased readiness potential—an electrocortical signal associated with the production of voluntary acts and strongly associated with a sense of responsibility [67]. While the link between sense of agency and moral identity could be rooted in feelings of responsibility, the positive relationship between sense of ownership and moral identity appears to be less straightforward and to contradict our initial hypothesis *H1*. However, this association may resemble the one found between enhanced body ownership and positive attributes. Consider, for example, the enfacement illusion [68], i.e. the inclusion of another person's face into an extended representation of one's own body. Crucially, this extension of the sense of body ownership is more likely to occur when the other person is considered as displaying desirable characteristics, such as niceness and physical attractiveness [69,70]. Therefore, it has been proposed that enhanced body ownership is observed in association with positive qualities, as doing so promotes the observer to attribute these qualities to themselves [71,72]. The positive relationship we found between sense of ownership and moral identity may be aligned to this, as increased ownership could facilitate the association between body and positive characteristics (like morality), that are consequently ascribed to the self.

Contrary to what we expected (see hypothesis *H2a*), our results suggest that sense of power is associated with an increase of both the sense agency and moral identity. In fact, in both studies, we found a positive relationship between sense of agency and moral identity, especially in participants who reported being able to influence others. Accordingly, previous research has shown that feeling powerful comes with a preference for deontological moral judgements [65], which rely on a style of moral thinking that focuses on abiding existing rules. Crucially, deontological judgements have been observed in association with enhancement of moral identity [73]. Considering all this, it is possible that, as sense of power increases, its effect adds to the positive relationship between agency and moral identity, which eventually becomes strengthened. While this is true for moral identity, our results show a lack of association between (im)moral behaviour, sense of agency and sense of power. This may find an explanation in the link between sense of power and opposite moral-thinking styles. A recent study [74] found that sense of power is associated with different styles of moral thinking, namely integration-oriented (which relies on affective and cognitive evaluations), deliberation-oriented (where the focus of the decision-making process is to maximize outcomes) and rule-oriented (focused on abiding by existing rules). Notably, the study suggests that these moral orientations may have opposite effects over moral judgements. Thus, while deliberation- and integration-oriented styles seem to favour outcome-based judgements, rule-orientation styles act in the opposite direction. Furthermore, deontological judgements seem to increase in association with integration-oriented styles, and are reduced by deliberation-oriented moral thinking. In the light of all this, we suggest that, during our task, the participants' sense of power may have activated different moral-thinking styles. It is possible that their opposing effects may have counteracted each other's bias towards (dis)honest behaviours, ultimately leading to the absence of any relationship between the senses of agency and power.

Our two studies highlighted a significant, direct relationship between moral disengagement and morality, where an increase of the former was associated with a reduction of moral identity. This is in line with research on moral disengagement, which shows that reliance on justification strategies is associated with less morality and a tendency to behave immorally [75,76]. Additionally, study 1 showed that, in line with our expectations (see *H2b*), the positive association of agency with moral identity remained significant only for participants with *high* (and not *low*) moral disengagement. However, we failed to replicate this result in study 2. One possible explanation may come from Bandura *et al*. [66], who argued that, when people are faced with multiple, alternative courses of action, they aim to behave in accordance with their moral standards. To achieve their goals while simultaneously avoiding immoral behaviours, people can employ self-censure mechanisms when making decisions. Self-sanctions appear to be strongly activated when the sense of responsibility over one's own actions and outcomes is high, and justifying future detrimental behaviour becomes difficult. Our data also suggest that sense of agency may activate these mechanisms, thus reducing (study 1) or possibly even counterbalancing (study 2) the impact that moral disengagement has over moral identity. While sense of agency and moral disengagement appear to be associated with each other and with moral identity, we found that their relationship to behaviour in a given task is not significant. One possible explanation for this is that anticipatory self-sanction mechanisms were activated once participants were presented with the opportunity of cheating in exchange for a higher pay-off. Therefore, self-sanctions may have prevented the use of moral disengagement strategies and consequently restrained deceptive behaviours.

Interestingly, our first study suggested that reward sensitivity could modulate the effect of sense of ownership over morality. Specifically, we observed that when participants felt little tempted by rewards, high sense of ownership was associated with (i) increased moral identity and (ii) more dishonesty during the STDT. However, the data we collected in study 2 did not confirm any of these results. While we do not have a ready explanation for this discrepancy, we speculate that this may have to do with the impact of COVID-19, which might have influenced the second but not the first study. It is possible, for example, that experiences related to the pandemic situation altered the role of specific components of BSC. For instance, a recent longitudinal study suggests that since the beginning of the pandemic, individuals are more attentive to signals coming from their internal organs compared to a time before the COVID-19 outbreak [77]. Such increase of attention towards visceral information could be beneficial to the maintenance of individuals' well-being. The significant, positive correlation we found between the sense of ownership of participants and how dangerous COVID-19 felt to them may be in keeping with our explanation. In accordance with this, Kim and Anderson found that attention towards rewarding stimuli is reduced when the threat of painful stimulation is presented to participants [78,79]. With this in mind, we argue that, the presence of an external threat such as that posed by pathogens may shape the way in which individuals reappraise their bodily signals (as a sign of threat and danger instead of need for reward). In other words, the sense of ownership may serve a protective function and prioritize information that benefits the health of individuals. In turn, the relevance of other types of information, such as those regarding the presence of rewards, may be reduced. Thus, in the pandemic context, sense of ownership and reward sensitivity could not predict the same (im)moral behaviour with respect to what we observed before COVID-19.

While it is our belief that the present study significantly expands the current knowledge on the role of body awareness into higher level cognitive functions, such as morality, we acknowledge some limitations. Although we tested two large samples of people, all of them were Italian-speaking, thereby reducing the generality of the results. The very cogent issue of whether culture can influence the way in which body ownership and agency—as well as moderating variables—impact morality and (dis)honest behaviour ought to be addressed in future studies. In particular, research should clarify whether cross-cultural differences in morality [80] and moral behaviours [81] could be traced to possible differences in awareness of bodily signals [82], or in the feelings of agency associated with pleasant or desirable outcomes [83].

We also observed that the proportion of lies during our STDT is smaller (between 10 and 17.16% of responses across our two studies) than that of plausible responses (i.e. 0–5 reported differences). Previous research showed that dishonesty increases with the size of monetary rewards [84]. Considering this, one possibility was that the participants would indiscriminately report the highest number of differences when faced with the opportunity of getting the maximal monetary pay-off. Instead, our data suggest that the reward condition did not over incentivize dishonesty, which appears to be in line with other studies. In fact, it has been shown that individuals generally refrain from lying for selfish purposes [85] and that this is more likely to occur when they are given only one shot (compared to multiple shots) at earning monetary rewards [86]. Similarly, Hilbig and Thielmann showed that the majority of their participants acted honestly independently of the size of monetary rewards, while only a small group of individuals showed consistent dishonesty [43].

Regarding dishonest behaviours during the STDT, one may also argue that participants who lied were the ones prioritizing the final pay-off at the expense of the task itself. While we acknowledge that these studies would have benefitted from a specific assessment of the engagement with the task, we looked at whether participants were focusing on the study by means of two attention-check questions, and by excluding those individuals who failed both. Additionally, it should be noted that giving participants the opportunity of violating task instructions (i.e. to report the number of differences they managed to find) and of cheating for higher monetary gain is indeed an essential feature of our version of the STDT. In fact, whenever participants reported more than the actual number of existing differences (i.e. five), we can infer that they did so because they read, understood and deliberately violated task instructions.

Crucially, we acknowledge that lying to participants in regard to the real number of differences between images (i.e. five) represents an important, ethical limitation of our two studies. However, and as also highlighted by Liu and colleagues for a similar task [47], the alternative, truthful version of our STDT would have to rely on images that differ for a certain number of details, a proportion of which is very difficult (but not impossible) to find. While this methodology would eliminate deception on the part of researchers, it would also prevent them from recognizing participants who lie and those who do not with absolute certainty, and therefore from studying dishonest behaviours in similar settings. Additionally, recent evidence suggests that the use of deception may not undermine individuals' trust nor affect their future performance in research investigations [87]. Considering all this, and despite calling for the development of more ethical approaches, we believe that the aims of the present studies justify giving participants incorrect information [88].

While we have investigated the relationships between morality and a relatively high number of factors, we acknowledge that other important variables may have been overlooked. For example, the present studies did not examine the possible moderating effect of gender in the relationship between different BSC components and morality. However, while it is possible that genders differ in terms of dishonest behaviour [84], we did not have any defined hypothesis regarding gender and the specific BSC components we have investigated.

In conclusion, we have demonstrated that moral identity is associated with different components of BSC. In fact, our results consistently show increased moral identity in association with enhanced senses of ownership and agency. Additionally, we observed that sense of power may have a modulating effect over agency. However, the role of other intervening variables, such as moral disengagement and reward sensitivity, appears to be less clear. Similarly, evidence regarding dishonest behaviours is not conclusive, as we could not confirm of the role of increased sense of ownership and reward sensitivity. Nonetheless, our study suggests that the moral identity of individuals could be strengthened through BSC. Policies investing in mindfulness trainings for the general population and in encouraging its regular practice may favour this process. In fact, exercising mindfulness meditation, especially through practices that revolve around focusing on bodily sensations, has been found to be positively associated with body awareness and with a more stable representation of one's body [89–94]. At the same time, future studies are needed to clarify which factors operate in synergy with BSC components during decision-making processes and whether and in what way COVID-19 could impact this process. Such knowledge could help develop specific training programmes, which would promote a reliance on mechanisms that could counterbalance the tendency to act immorally.

## Data Availability

The code for replication of all analysis is available at doi:10.17632/84tz3jkhr3.2 [95]. The full dataset for replication of the results presented here will be made available to anyone upon personal communication with the authors of this study and only upon agreeing to use the dataset exclusively for replication purposes. In fact, 32 of the participants who took part in study 1 (4.86% of the final sample) and 45 in study 2 (6.91% of the final sample) did not give their consent to the use of their data for testing new hypotheses. As a consequence, these participants have been excluded from the dataset for investigating new hypotheses, which is retrievable at the following: doi:10.17632/84tz3jkhr3.2 [95]. Data is also to be found in the electronic supplementary material [96].
